# Trace Elements Contamination and Human Health Risk Assessment in Drinking Water from the Agricultural and Pastoral Areas of Bay County, Xinjiang, China

**DOI:** 10.3390/ijerph13100938

**Published:** 2016-09-23

**Authors:** Muyessar Turdi, Linsheng Yang

**Affiliations:** 1 Key Laboratory of Land Surface Pattern and Simulation, Institute of Geographical Sciences and Natural Resources Research, Chinese Academy of Sciences, 11 A Datun Road, Beijing 100101, China; muys.14b@igsnrr.ac.cn; 2 University of Chinese Academy of Sciences, Beijing 100049, China

**Keywords:** trace elements, human risk assessment, drinking water, agriculture areas, pastoral areas, Xinjiang

## Abstract

Tap water samples were collected from 180 families in four agricultural (KYR: Keyir, KRW: Kariwak, YTR: Yatur, DW: Dawanqi) and two pastoral areas (B: Bulong and Y: Yangchang) in Bay County, Xinjiang, China, and levels of seven trace elements (Cd, Cr, As Ni, Pb, Zn, Se) were analyzed using inductively-coupled plasma mass spectrometry (ICP-MS) to assess potential health risks. Remarkable spatial variations of contamination were observed. Overall, the health risk was more severe for carcinogenic versus non-carcinogenic pollutants due to heavy metal. The risk index was greater for children overall (Cr > As > Cd and Zn > Se for carcinogenic and non-carcinogenic elements, respectively). The total risk index was greater in agricultural areas (DW > KYR > YTR > KRW > B > Y). Total risk indices were greater where well water was the source versus fountain water; for the latter, the total health risk index was greater versus glacier water. Main health risk factors were Cr and As in DW, KYR, YTR, KRW, and B, and Zn, Cr, and As in the Y region. Overall, total trace element–induced health risk (including for DW adults) was higher than acceptable (10^−6^) and lower than priority risk levels (10^−4^) (KYR, YTR, KRW, Y, and B). For DW children, total health risk reached 1.08 × 10^−4^, higher than acceptable and priority risk levels (10^−4^).

## 1. Introduction 

Trace elements exist widely in specific concentrations in the natural environment [1]. With the development of the economy and society human activities, such as mining, smelting, and processing, have allowed more trace elements to enter the atmosphere, water, and soil, thus resulting in serious environmental pollution [2]. Pollution from trace elements has become the main source of global environmental pollution. Their emission into the environment is harmful not only to ecosystems, but also poses a threat to human health because of refractory characteristics of bioaccumulation [3]. Although essential trace elements are critical for life processes and sustainability, they are only needed at the trace level [4]. Excess intake of essential trace elements in drinking water may lead to adverse health effects [5,6]. In particular, elements such as cadmium, chromium, arsenic, and lead have significant biological toxicity and are harmful to human health [7,8,9], for example. Cadmium mainly accumulates in the human hepatic system and kidneys, disturbing estrogen secretion, and is also carcinogenic [10,11]. Chromium is one of the trace elements with the strongest biological toxicity, and can cause people to develop liver and skin cancer. Lead disturbs gonad secretion [12]. Copper is a necessary trace element for the human body, but if its concentration goes above the necessary amount, it is harmful to the liver, kidney, digestive system, and brain [13,14]. Zinc can cause serious damage to the digestive system, nervous system, and blood system [9,15]. Selenium deficiency can accelerate the body’s aging processes and may lead to cancer, cardiovascular disease, diabetes, Kaschin–Beck disease, and increasing numbers of other diseases [9,16]. 

Recently, with the development of agricultural production, there has been broad application of the chemical fungicides [17]. Residual pesticides circulate through the atmosphere, water, soil, and biosphere, but some of the pesticides are vestigial in surface water and groundwater [18,19]. They still cannot be completely removed through the process of modern drinking water treatment (sediment, filter, disinfection), so the risks that are caused by these vestigial pesticides in drinking water are also on the rise [20]. The typical source of the drinking water in rural areas in China is well water, which is easily affected by the environmental pollution factors [15]. There have been some serious phenomena in most of these rural areas, such as drinking water resources being below normal standards and, often, heavily polluted [21]. Further problematic phenomena include arbitrary disposal of wastewater from township enterprises along with substandard water treatment, while the trace elements in the drinking water exceed the standard [22]. These problems threaten the safety of the drinking water for rural populations. It has become an urgent need to discover how to improve the condition of the water supply in order to ensure the safety of drinking water for the vast numbers of people in China’s rural areas. 

Even though China has made substantial efforts to improve the standard of drinking water, the constraints of limited technical expertise, as well as inadequate laboratory facilities and resources, limits the ability to monitor water quality to only a few locations or administrative areas in Xinjiang, China. Moreover, rural and remote areas, where drinking water contamination may be more severe, are often excluded from monitoring [15,23]. Consequently, regional-scale risk assessments are often unavailable, and information on the extent of trace contamination and the total population at risk is largely unknown [24]. Therefore, the comparison of the trace element levels and their effect on the health of the local people in areas with different sources of drinking water is important, particularly when taking into account the topography from the different typical agricultural and pastoral areas in Bay County, Xinjiang, China. For this paper, we analyzed the drinking water from 180 households in typical agricultural (KYR: Keyir, KRW: Kariwak, YTR: Yatur, DW: Dawanqi) and animal husbandry regions (B: Bulong and Y: Yangchang). We compared the trace elements Ni, Zn, Se, Cr, As, Cd, and Pb in the different regions for content distribution and preliminary evaluation. These trace element–associated health risk comparisons in the drinking water of different regions were based on China’s health standards for drinking water (GB574-2006) as well as the health risk model recommended by the United States Environmental Protection Agency (EPA). It was, thus, the health standards of both China and the U.S. that provided the basis for determining the trace element exposure level in drinking water that poses a threat to human health, along with providing the scientific basis for environmental risk management.

## 2. Materials and Methods

### 2.1. Study Area

Bay County is in the southwest of the Xinjiang Uyghur Autonomous Region. It is located in the middle of the Tianshan Mountains, in the basin of the northern margin of the Queletagh Mountains and the upstream region of the Weigan River; specifically, it is between 80°37′39″–83°02′25″ E and 41°24′08″–42°38′52″ N [25]. There is one county town (Bay County town), and there are three building towns (Tirek, Sairam, Chaierqi), ten villages (Keyir, Kezili, Tuokesun, Yaturi, Kanqi, Bulong, Miqiki, Ombax, Daqao, Kariwahi), two state farms (Dawanqi, Yakeriki), one breeding farm, and one agricultural experiment station in this county. The northwest terrain of Bay County is low, but its southeast terrain is high. To the north of Bay County is the grand trunk of the Tianshan Mountains. It is more than 4500 m above sea level, with plentiful snow and many glaciers which can be found year round. Bay County is framed by the Queletagh Mountains to the west and south; it is between 1180 m and 1400 m above sea level. 

The total stream of the river, surface water, and total groundwater storage available for exploitation in the Bay County town are respectively 28.1 million m^3^, 31.0 million m^3^, 27.80 million m^3^, and 11.00 million m^3^ [26]. Due to the temperate continental climate, there are various water resources such as Tianshan glacier water, surface river water and spring water, underground confined water and phreatic water, atmospheric precipitation, and artificial lake water. There are five rivers in Bay County; from west to east, there are the Muzat River, Kapsilan River, Teliwiqik River, Karsu River, and Kezir River, all of which are important resources for life and production [27]. The agricultural irrigation water is rich, and the county’s water consumption is 14.55 million m^3^. There is also spring water that is formatted by the upstream riverbed leakage on the hillside [5]. The DW, YTR, KYR, and KRW belong to the agricultural region and the Y and B belong to the pastoral area. The drinking water source of the DW agricultural region is the well water, and the sources of the drinking water for the YTR and KYR agricultural regions are the fountain water. The drinking water source of the KRW agricultural is mountain water from melting glaciers. The Y pastoral region belongs to the plateau region where the source of the drinking water is mountain water from melting glaciers. The drinking water source of the pastoral B region is the fountain water.

### 2.2. Sample Collection

**Figure 1:**
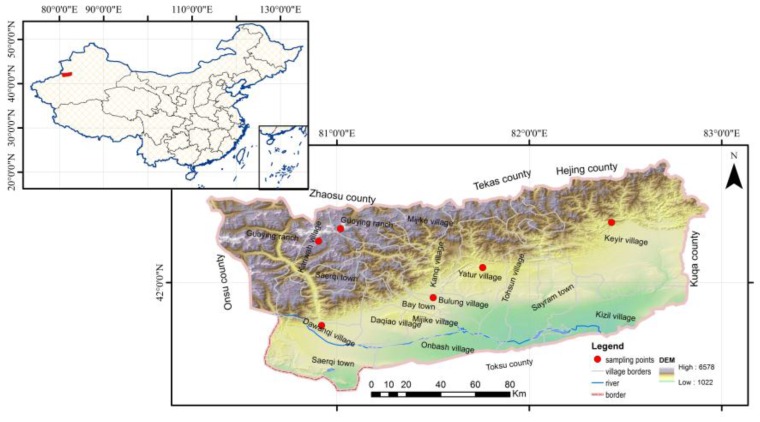
The study area.

In July 2015, according to the range of the region’s water supply and population distribution, we used multi-stage random sampling methods and collected samples of the tap drinking water of 180 families from the water containers of every family from the typical agricultural (Keyir, Kariwah, Dawanqi, Yaturi) and natural pastoral areas (Bulong, Yangchang) of Bay County (Figure 1). Thereafter, about 1 L of water was collected in a plastic container and 10 mL of nitric acid was added for preservation. The acidified drinking water samples were stored at 4 °C and analyzed within seven days of collection. We collected and preserved the water samples in accordance with the GB/T5750.2-2006 Standard Test for Drinking Water.

### 2.3. Chemicals and Reagents

Ultra-pure analytical-grade nitric acid with a concentration of 65% was purchased from Guangzhou Chemical Reagent Factory (Guangzhou, China). Standard solutions of the trace elements (Cr, As, Cd, Ni, Zn, Se and Pb) were obtained from the National Institute of Metrology of China (NIM, Beijing, China). 

#### Instrumental Analysis and Quality Control

Each 10-mL water sample was acidified with 100 μL of nitric acid, and the concentrations of trace elements were determined using Agilent 7700x inductively-coupled plasma mass spectrometry (ICP-MS). Optimized instrumental parameters are listed in Table 1. We used ICP-MS and tested the elements (Cr, As, Cd, Ni, Zn, Se, and Pb).

Standard curve solutions of the trace elements were prepared with 1% of nitric acid ranging from 0.002 to 0.500 μg/L for Se; 0.005 to 3.000 μg/L for Cd; 0.030 to 3.500 μg/L for Cr, As, and Pb; 0.100 to 4.000 μg/L for Ni; and 0.500 to 3000 μg/L for Zn, respectively. The calibration curve regression coefficients (*r*^2^) for individual elements were all above 0.9995. We used the internal standard solutions for checking the signal drift during instrumental analysis. To authenticate the stability of the detector response, a moderate concentration of multi-element standard solution was analyzed with each batch of five samples, and relative standard deviation was less than 10%. Recoveries of the trace elements were obtained by spiking standard solutions to water samples at two levels (2 μg/L and 10 μg/L), and the spiked samples were also subjected to the same procedure used for the samples. Recoveries for all of the elements in the present study were between 83% and 104%. We analyzed the procedural blank and reagent blank with each batch of five samples to check for potential contamination in the laboratory. We also defined the limit of detection (LOD) and limit of quantification (LOQ) as three and ten times the relative standard deviation for 21-reagent blank analysis, respectively. 

### 2.4. Data Analysis

In this study we used descriptive statistics, the Kruskal-Wallis test (StatSoft, Inc., Tulsa, OK, USA), and a human health risk assessment method [28,29,30]. 

#### 2.4.1. Human Health Risk Assessment

According to the toxicological effect, the health risks of exposure to pollutants include carcinogenic and non-carcinogenic risks. The evaluation models of health risk assessment as recommended by the U.S. Environmental Protection Agency (EPA) were also used in this study.

*ADD_j_* is the dose of carcinogenic chronic chemical pollutants by everyday drinking, with units of [mg·(kg·d)^−1^]. It can be calculated by the following formula:

(1) $$ADD_{j}=\frac{C\times IR\times ED\times EF}{BW\times AT}$$

In Equation (1), *C* is the average concentration of the chemical pollutants (mg·L^−1^); *IR* is for the average daily water consumption (2.2 L for adults’ and 1 L for children’s average daily water consumption); *ED* is the exposure cycle (70 a); *EF* is the exposure frequency (365 days); *BW* is the adult’s body weight (adults, 70 kg; children, 25 kg); *AT* is the life time (365 days × 70 years).

*PAD* is the adjustment dose of non-carcinogenic chronic chemical pollutants by the drinking water, and has units of [mg·(kg·d)^−1^]. It can be calculated by this formula:

(2) $$PAD_{ig}=\frac{RfD_{ig}}{10}$$

In Equation (2), *RfD* is the reference dose of the non-carcinogenic chronic chemical pollutants, with units of [mg·(kg·d)^−1^]. In this study, the safety factor value is 10.

The risk model for health hazards caused by carcinogenic chemical pollutants is expressed as a risk of cancer caused by exposure to a carcinogen, which is more than a normal level of cancer. It is generally believed that the carcinogenic compound in water has a linear relationship with its concentration. The evaluation formula is as follows:

(3) $$\begin{array}{c} R_{ig}^{c}=\frac{1\times \text{exp}(ADD_{ig}\times q_{ig})}{Y}\\ R^{c}=\sum_{J=1}^{J}R_{j}^{c} \end{array}$$

In Equation (3), *R* is the annual cancer risk that results from the carcinogenic chemical pollutants which are ingested by the person (a^−1^); *q* is the dose of the chemical pollutant carcinogenic intensity coefficient (mg·(kg·d)^−1^)^−1^; *Y* is the average life expectancy of 70 years.

The risk model for health hazards caused by non-carcinogenic chemical pollutants is calculated by the following formula:

(4) $$\begin{array}{c} R_{ig}^{n}=\frac{ADD_{ig}\times 10^{-6}}{PAD_{ig}\times Y}\\ R^{D}=\sum_{K=1}^{K}R_{k}^{c} \end{array}$$

In Equation (4), *R* is the annual health risk resulting from the non-carcinogenic chemical pollutants ingested by the person (a^−1^).

When we calculate with multiple substances and risk types, we first calculate all of the carcinogenic and non-carcinogenic risks, and then find the sum. Generally, we do not consider the synergy reaction and the antagonism reaction:

(5) $$R_{sum}=R^{c}\pm R^{c}$$

#### 2.4.2. Parameter Values 

The International Association for Cancer Research (IARC) and the World Health Organization (WHO) comprehensively evaluated the chemical pollutants’ carcinogenicity and then formulated the classification system. Cd, Cr, and As belong to the class of carcinogenic chronic chemical pollutants and their reference dose value and chemical pollutants carcinogenic intensity coefficients are indicated in Table 2. Ni, Zn, Se, and Pb belong to the non-carcinogenic chronic chemical pollutants and the values of the reference doses of the non-carcinogenic chronic chemical pollutants are shown in Table 2 [8]. The maximum risk acceptance risk level and negligible level of some organizations are listed in Table 3 [31].

## 3. Results 

### 3.1. Trace Element Concentrations in Drinking Water

Compared to China’s drinking water standard (GB5749-2006), virtually all trace elements in the drinking water in the present study of the six rural areas could meet the regulation requirements (Table 4). The water pH ranges from 6.0–7.12. There is no significant difference (*p* ≥ 0.05) in the water pH levels among the sampling points. The water pH was within the range recommended by WHO (6.5–8.5) for all sampling points. All trace elements were detected in tap water, and the concentrations varied greatly.

**Figure 2:**
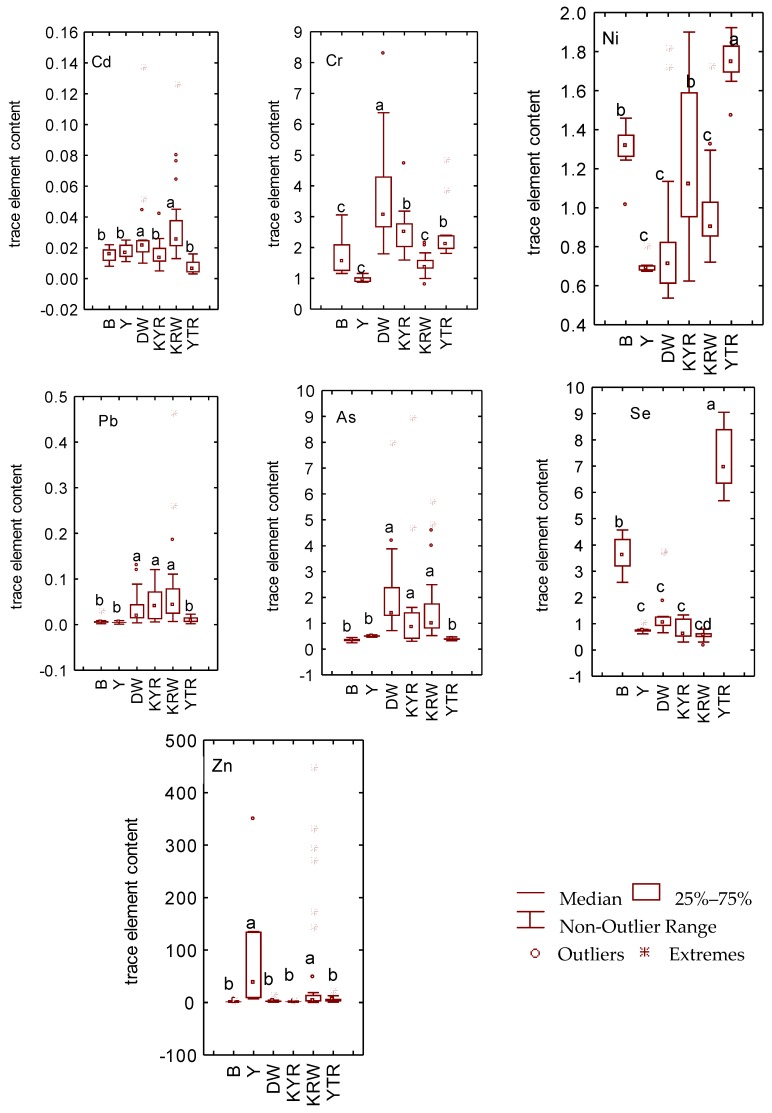
The trace element concentrations in the drinking water of Bay County. Boxes with different letters were significantly different at *p* < 0.05. Significance was determined by nonparametric comparison (Kruskal-Wallis test. DW: Dawanqi; KYR: Keyiri; KRW: Kriwahi; Y: Yangchang; YT: Yaturi; B: Bulong.

It is evident that the mean content of Cr in Dawanqi is higher than that of other regions; the Cr mean content of the Kyiri and Yaturi is similar and about 2.5 μg/L; the mean content of Cr in the Bulong, Kariwahi, and Yangchang is similar and about 1–1.7 μg/L (Figure 2). The mean content of Cd in Keyir, Yatur, Bulung and Yangchang is similar with the mean value of 0.007–0.017 μg/L, but all are significantly lower than that of Dawanqi and Kariwahi with mean values of about 0.03 μg/L. There is no significant difference between Dawanqi, Karwahi, and Keyir in As content, which is about 1.5–2.3 μg/L, however these regions have significantly higher levels of As compared to Yatur, Bulung, and Yangchang which have similar amounts of As (0.3–0.4 μg/L). The Zn content of the Karwahi and Yangchang regions is higher than that of the other regions, and the mean values are 56.23 μg/L and 96.18 μg/L, respectively. However, the other regions have relatively low levels of Zn content and have little variance between one another. There is a slight difference in Pb content between Dawanqi, Kariwahi, and Keyir regions, with the mean value lower than 0.1 μg/L. However, these regions have relatively higher levels of Pb than that of other regions (YTR, Y, B), which share no significant difference in Pb. The mean content of Ni in the Dawanqi and Kerwahi regions is very similar, at about 0.9 μg/L; the mean content of Ni in the Keyiri and Bulung regions is also similar at about 1.2 μg/L. It is noteworthy that the highest level of Ni content is found in the Yaturi region, while the Yangchang has the lowest levels of Ni, with an average content of 0.7 μg/L. The mean content of Se in the Keyir, Kerwahi, and Yangchang regions is almost the same, at about 0.55–0.7 μg/L; the order of the Se content of the other three regions is Yatur > Bulung > Dawanqi. 

### 3.2. Human Health Risk Assessment

According to the evaluation of health risk assessment models recommended by the U.S. EPA, we assessed the level of health hazard risks for the population in four agricultural areas and two pastoral areas of the Bay County in Xinjiang, China caused by trace elements ingested orally through drinking water. Two population groups were considered: adults and children.

**Figure 3:**
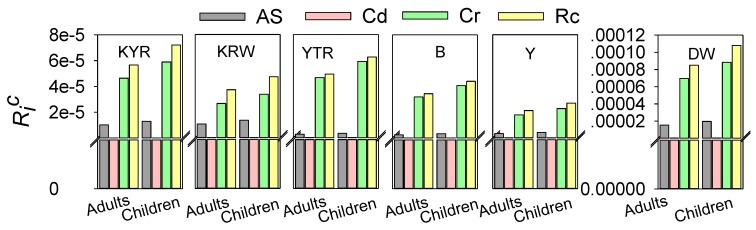
The value of the carcinogenic health risks assessment of the different regions of Bay County. KYR: Keyir, KRW: Kariwak, YTR: Yatur, DW: Dawanqi, Y: Yangchang, B: Bulong.

**Figure 4:**
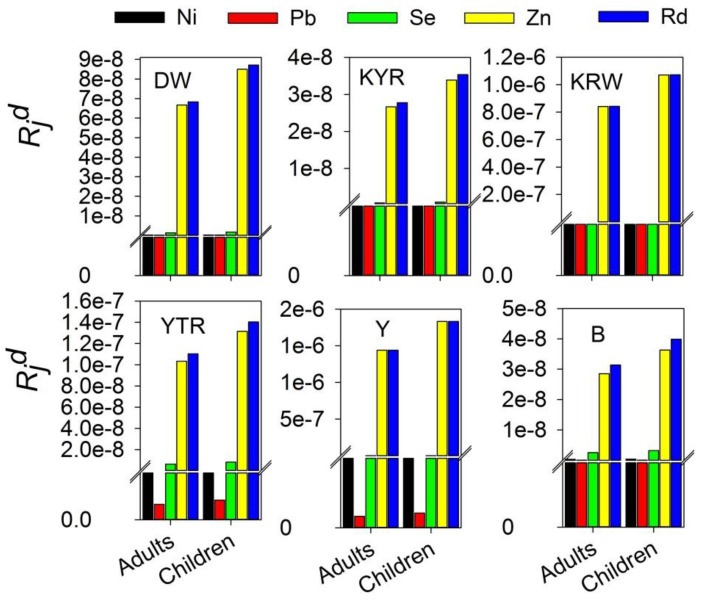
The value of the human health risk assessment induced by non-carcinogenic chemical pollutants of the different regions of Bay County. KYR: Keyir, KRW: Kariwak, YTR: Yatur, DW: Dawanqi, Y: Yangchang, B: Bulong.

**Figure 5:**
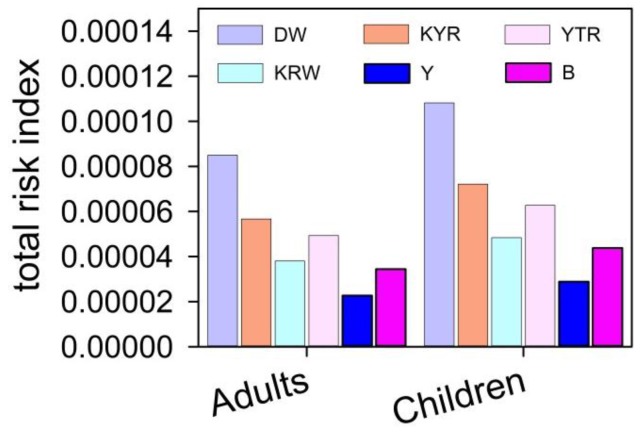
The total value of the human health risk induced by both the carcinogenic and non-carcinogenic chemical pollutants of the different regions of Bay County. KYR: Keyir, KRW: Kariwak, YTR: Yatur, DW: Dawanqi, Y: Yangchang, B: Bulong.

The median values of incremental lifetime health risks induced by carcinogenic metals of the DW agriculture region were estimated to be 6.95123 × 10^−5^, 1.53707 × 10^−5^, and 8.74299 × 10^−8^ in adults; and 8.8411 × 10^−5^, 1.95598 × 10^−5^, and 1.1127 × 10^−8^ in children for Cr, As, and Cd, respectively (Figure 3). The total health risks induced by carcinogenic metals of the DW agriculture region are 8.50 × 10^−5^ for adults and 1.08 × 10^−4^ for children, while the median values of non-carcinogenic chemical pollutants health hazard risk were estimated to be 6.6667 × 10^−8^, 1.94 × 10^−10^, 1.30007 × 10^−10^, 1.32 × 10^−9^ in adults; and 8.5005 × 10^−8^, 2.47 × 10^−10^, 1.65463 × 10^−10^, and 1.68 × 10^−9^ in children for Zn, Ni, Pb, and Se, respectively (Figure 3 and Figure 4). The total non-carcinogenic chemical pollutants health hazard risk of the DW agriculture region is 6.83 × 10^−8^ for adults and 8.71 × 10^−8^ for children (Figure 4). The total health risks induced by both the carcinogenic chemical pollutants and the non-carcinogenic chemical pollutants are 8.50387 × 10^−5^ for adults and 1.0817 × 10^−4^ for children (Figure 5).

The median values of incremental lifetime health risks induced by carcinogenic metals of the KYR agriculture region were estimated to be 4.64 × 10^−5^, 1.02 × 10^−5^, and 4.43037 × 10^−8^ in adults; and 5.9 × 10^−5^, 1.3 × 10^−5^, and 5.63864 × 10^−8^ in children for the Cr, As, and Cd, respectively (Figure 3). The total health risks induced by carcinogenic metals of the KYR agriculture region are 5.66 × 10^−5^ for adults and 7.21 × 10^−5^ for children (Figure 3), while the median values of the non-carcinogenic chemical pollutant health hazard risk were estimated to be 2.66 × 10^−8^, 2.74247 × 10^−10^, 1.45824 × 10^−10^, and 7.05426 × 10^−10^ in adults; and 3.39 × 10^−8^, 3.49042 × 10^−10^, 1.85594 × 10^−10^, and 8.97815 × 10^−10^ in children for Zn, Ni, Pb, and Se, respectively (Figure 4). The total non-carcinogenic chemical pollutant health hazard risk of the KYR agriculture region is 2.78 × 10^−8^ for adults and 3.53 × 10^−8^ for children (Figure 4). The total health risks induced by both the carcinogenic chemical pollutants and the non-carcinogenic chemical pollutants are the 5.67134 × 10^−5^ for adults and 7.21532 × 10^−5^ for children (Figure 5).

The median values of incremental lifetime health risks induced by carcinogenic metals of the KRW agriculture region were estimated to be 2.65 × 10^−5^, 1.06 × 10^−5^, and 9.22881 × 10^−8^ in adults; and 3.36563 × 10^−5^, 1.35248 × 10^−5^, and 1.17458 × 10^−7^ in children for the Cr, As, and Cd, respectively (Figure 3). The total health risks induced by carcinogenic metals of the KRW agriculture region are 3.72 × 10^−5^ for adults and 4.73 × 10^−5^ for children (Figure 3). The median values of the non-carcinogenic chemical pollutants health hazard risk were estimated to be 8.4159 × 10^−7^, 2.13204 × 10^−10^, 2.30807 × 10^−10^, and 4.98694 × 10^−10^ in adults; and 1.0711 × 10^−6^, 2.71351 × 10^−10^, 2.93754 × 10^−10^, and 6.34701 × 10^−10^ in children for Zn, Ni, Pb, and Se, respectively (Figure 4). The total non-carcinogenic chemical pollutants health hazard risk of the KRW agriculture region is 8.43 × 10^−7^ for adults and 1.07 × 10^−6^ for children; the total health risks induced by both the carcinogenic chemical pollutants and the non-carcinogenic chemical pollutants are 3.80 × 10^−5^ for adults and 4.84 × 10^−5^ for children (Figure 5).

The median values of incremental lifetime health risks induced by carcinogenic metals of the YTR agriculture region were estimated to be 4.65592 × 10^−5^, 2.63639 × 10^−5^, and 2.1362 × 10^−8^ in adults; and 5.92308 × 10^−5^, 3.3553 × 10^−5^, and 2.71188 × 10^−8^ in children for the Cr, As, and Cd, respectively (Figure 3). The total health risks induced by carcinogenic metals of the YTR agriculture region are 4.93 × 10^−5^ for adults and 6.26 × 10^−5^ for children (Figure 3). The median values of non-carcinogenic chemical pollutant health hazard risks were estimated to be 1.03 × 10^−7^, 3.91892 × 10^−10^, 3.30321 × 10^−11^, and 6.50616 × 10^−9^ in adults; and 1.31 × 10^−7^, 4.99 × 10^−10^, 4.20 × 10^−11^, and 8.28 × 10^−9^ in children for Zn, Ni, Pb, and Se, respectively (Figure 4). The total non-carcinogenic chemical pollutant health hazard risk of the YTR agriculture region is 1.10 × 10^−7^ for adults and 1.40 × 10^−7^ for children. The total health risks induced by both the carcinogenic chemical pollutants and the non-carcinogenic chemical pollutants are 4.94 × 10^−5^ for adults and 6.27 × 10^−5^ for children (Figure 5).

The median values of incremental lifetime health risks induced by carcinogenic metals for the Y natural pastoral areas were estimated to be 1.79 × 10^−5^, 3.29 × 10^−6^, and 4.79 × 10^−8^ in adults; and 2.277 × 10^−5^, 4.19081 × 10^−6^, and 6.1 × 10^−8^ in children for the Cr, As, and Cd, respectively (Figure 3). The total health risks induced by carcinogenic metals of the Y natural pastoral areas are 2.12 × 10^−5^ for adults and 2.70 × 10^−5^ for children (Figure 3). The median values of non-carcinogenic chemical pollutants health hazard risk were estimated to be 1.4396 × 10^−6^, 1.58565 × 10^−10^, 1.65695 × 10^−11^, and 7.08041 × 10^−10^ in adults; and 1.83 × 10^−6^, 2.0181 × 10^−10^, 2.10884 × 10^−11^, and 9.01143 × 10^−10^ in children for Zn, Ni, Pb, and Se, respectively (Figure 4). The total non-carcinogenic chemical pollutants health hazard risk of the Y natural pastoral areas is 1.44 × 10^−6^ (adults) and 1.83 × 10^−6^ (children). The total health risks induced by both the carcinogenic chemical pollutants and the non-carcinogenic chemical pollutants are 2.26 × 10^−5^ for adults and 2.88 × 10^−5^ for children (Figure 5).

The median values of incremental lifetime health risks induced by carcinogenic metals of the B natural pastoral areas were estimated to be 3.19392 × 10^−5^, 2.39272 × 10^−6^, and 4.19374 × 10^−8^ in adults; and 4.06375 × 10^−5^, 3.35532 × 10^−6^, and 5.33749 × 10^−8^ in children for Cr, As, and Cd, respectively (Figure 3). The total health risks induced by carcinogenic metals of the B natural pastoral areas are 3.44 × 10^−5^ for adults and 4.40 × 10^−5^ for children (Figure 3). The median values of non-carcinogenic chemical pollutants health hazard risks were estimated to be 2.85 × 10^−8^, 2.94 × 10^−10^, 2.36516 × 10^−11^, and 2.51429 × 10^−8^ in adults; and 3.63 × 10^−8^, 3.75 × 10^−10^, 3.0102 × 10^−11^, 3.2 × 10^−9^, and in children for Zn, Ni, Pb, and Se, respectively (Figure 4). The total non-carcinogenic chemical pollutants health hazard risks of the B natural pastoral areas are 3.14 × 10^−8^ (adults) and 3.99 × 10^−8^ (children). The total health risks induced by both the carcinogenic chemical pollutants and the non-carcinogenic chemical pollutants are 3.44052 × 10^−5^ for adults and 4.3776 × 10^−5^ for children (Figure 5).

## 4. Discussion

We can see that the trace elements in the tap water of the six regions were very different with regard to the content coefficient of variation. We find that while the people in the same region drink tap water from the same source, there are significant differences between the containers used for saving water, as well as differences in the materials used for the pipes through which the tap water passes. Additionally, because people have different habits for getting the drinking water from the tap and putting it into containers, the result is a significant difference in the heavy metal content of the water.

In recent years, the issue of drinking water polluted by trace elements had received keen attention around the world, especially in developing countries. For instance, a previous study assessed tap water quality in one of the villages of Gao Ming Foshan City, Guangdong Province [15]. In that particular study, the mean concentrations of individual essential trace elements were 0.775 μg/L, 4 μg/L, 0.06 μg/L, 0.375 μg/L, and 0.27 μg/L, for As, Cr, Cd, Pb, and Se, respectively. The concentrations of As and Se in our research were higher than those in their study, while the other elements maintained lower levels than those of Foshan, especially for Pb. 

We find some similar phenomena in the human health risk assessment of the six regions. Over all six regions, the value of Cr is highest among the health hazard risk index due to carcinogenic chemical pollutants, and the value of Zn is highest among the health hazard risk index due to non-carcinogenic chemical pollutants. The health hazard risk index caused by carcinogenic chemical pollutants is greater than that caused by non-carcinogenic chemical pollutants, and is consistent with similar studies performed in China, which concluded that the carcinogenic chemical pollutants health hazard cancer risk is more severe than the non-carcinogenic chemical pollutants health hazard risk due to heavy metals in drinking water. The size of the risk index of the carcinogenic chemical trace elements of the six regions in order is Cr > As > Cd, and that of the risk index of the non-carcinogenic chemical trace elements of the study areas is Zn > Se. Both health hazard risk indices are greater for children than for adults. 

In the six regions, comparing the median total cancer risk values associated with exposure to carcinogenic trace elements via consumption of drinking water, the size of the sum risk index is DW (adults) > KYR (adults) > YTR (adults) > KRW > (adults) > B (adults) > Y (adults); DW (children) > KYR (children) > YTR (children) > KRW (children) > B (children) > Y (children). Comparing the median values of incremental lifetime total non-cancer risks induced by non-carcinogenic metals, the size of the sum of the risk index is Y (adults) > KRW (adults) > YTR (adults) > DW (adults) > KYR (adults) > B (adults); Y (children) > KRW (children) > YTR (children) > DW (children) > B (children) > KYR (children). Comparing the median values of the incremental lifetime total health risk induced by trace elements in drinking water, the size of the sum risk index is DW (adults) > KYR (adults) > YTR (adults) > KRW (adults) > B (adults) > Y (adults); DW (children) > KYR (children) > YTR (children) > KRW (children) > B (children) > Y (children). The incremental lifetime total health risk induced by trace elements in the drinking water of typical agricultural regions is greater than in the natural pastoral areas.

Since the incremental lifetime non-cancer risk was less than 10^−6^, the risk induced by these non-carcinogenic elements in drinking water is considered inconsequential for people in the following regions: DW, KYR, YTR, KRW, and B. The trace Cd cancer risk was also less than 10^−6^, so the risk that is induced by Cd in drinking water is considered inconsequential for populations of DW, KYR, YTR, KRW, and B, and the main factors of cancer risk for these populations were Cr and As. Since the incremental lifetime non-cancer risks induced by Ni, Pb and Se were less than 10^−6^, the risks induced by Ni, Pb and Se elements in drinking water are considered inconsequential for those in the Y region, with the main factor of the non-cancer risk for the Y population was Zn. The cancer risks of carcinogenic trace Cd were also less than 10^−6^ for both Y populations, so the risks that are induced by the Cd in drinking water are considered inconsequential, with the main cancer risks for these populations being Cr and As. The total value of incremental lifetime health risks induced by the seven trace elements for the DW adult population reached 8.51 × 10^−5^, which is higher than the acceptable risk level (10^−6^) and lower than the priority risk level (10^−4^), while the DW children’s population reached 1.08 × 10^−4^, which is higher than both the acceptable risk level and the priority risk level (10^−4^). The total value of incremental lifetime health risk induced by the seven trace elements for the KYR adult population reached 5.67 × 10^−5^, and the KYR children’s population reached 7.21 × 10^−5^, with both of them being higher than the acceptable risk level (10^−6^) and lower than the priority risk level (10^−4^). The total value of incremental lifetime health risk induced by the seven trace elements for the KRW adult population reached 3.80 × 10^−5^, and the KRW children’s population reached 4.84 × 10^−5^, with both of them being higher than the acceptable risk level (10^−6^) and lower than the priority risk level (10^−4^). The total value of incremental lifetime health risk induced by the seven trace elements for the YTR adult population reached 4.94 × 10^−5^, and the YTR children’s population reached 6.27 × 10^−5^, with both of them being higher than the acceptable risk level (10^−6^) and lower than the priority risk level (10^−4^). The total value of incremental lifetime health risk induced by the seven trace elements for the Y adult population reached 2.27 × 10^−5^, and the Y children’s population reached 2.88 × 10^−5^, with both of them being higher than the acceptable risk level (10^−6^) and lower than the priority risk level (10^−4^). The total value of incremental lifetime health risk induced by the seven trace elements for the B adult population reached 3.44 × 10^−5^, and the B children’s population reached 4.40 × 10^−5^, with both of them being higher than the acceptable risk level (10^−6^) and lower than the priority risk level (10^−4^). This suggests a high potential for health risks from the trace elements in drinking water, implying that this class of elements requires dedicated attention.

The topography of the six regions is different and, thus, so is the source of the heavy metals. The drinking water source of the DW agricultural region is the well water, and the source of drinking water for the YTR and KYR agricultural regions, as well as the pastoral B region is fountain water. The drinking water source of the KRW agricultural region and the Y pastoral region is mountain water from melting glaciers.

The DW, YTR, KYR, and KRW regions are those where the agricultural production activities are relatively broad; hence, trace elements from pesticides are readily found in their agriculture. Coal and iron mines operate in the Y Plateau regions, so trace elements from these industries are readily found in the drinking water; thus, Zn is the main factor of the non-cancer risks for the Y population. 

## 5. Conclusions

All trace elements in the drinking water of the six regions in the present study could meet the regulatory requirements.

The risk indices of the carcinogenic and non-carcinogenic chemical trace elements in the six regions are in the order of Cr > As > Cd and Zn > Se, respectively. Both the non-carcinogenic and carcinogenic chemical pollutant health hazard risk indices for children are greater than for adults. 

The carcinogenic chemical pollutants health hazard cancer risk from trace elements is more severe than that of non-carcinogenic chemical pollutants in the drinking water of the six regions.

The total risk indices of the adults and children of the six areas are in the order of DW > KYR > YTR > KRW > B > Y. The total health risk for both the adults and children of the agricultural areas (KYR, YTR, KRW, and DW) is greater than in the pastoral areas (Y and B). The total risk indices in regions where the drinking water source is well water are greater than the total health risk indices in regions where the source of the drinking water is fountain water; the source of the fountain water regions’ total health risk indices is greater than in regions where the source is mountain water from melting glaciers.

The main factors of the cancer risks in the DW, KYR, YTR, KRW, Y, and B areas are Cr and As. The non-carcinogenic chemical pollutant health hazard risk and the Cd cancer risk in the drinking water are considered inconsequential for the people of the DW, KYR, YTR, KRW, and B regions. The Zn in the drinking water is considered the main non-carcinogenic chemical pollutant health hazard risk factor for the Y population.

The total health risk for both adults and children induced by the trace elements for KYR, YTR, KRW, Y, and B regions is higher than the acceptable risk level (10^−6^) and lower than the priority risk level (10^−4^). The total health risk induced by the trace elements for the DW adult is higher than the acceptable risk level (10^−6^) and lower than the priority risk level (10^−4^), while the total health risk for the population of DW children reached 1.08 × 10^−4^, which is higher than both the acceptable and priority risk levels (10^−4^).

## Figures and Tables

**Table 1:** Optimized instrumental parameters of inductively-coupled plasma mass spectrometry.

Parameters	Values
RF forward power	1500 W
Plasma gas flow rate	15 L/min
Carrier gas flow rate	0.80 L/min
Auxiliary gas flow rate	0.40 L/min
Nebulizer pump	0.10 rps
S/C tempreture	2 °C
Sample depth	8.0 mm
Resolution, 10% peak height	0.6–0.7 amu
Number of sweep	2
Collision cell helium flow rate	5.0 L/min
Cell entrance	−40 V
Cell exit	−60 V
Octopole bias	−18 V
Quadrupole bias	−15 V
Online internal standard (concentration)	45 Sc and 72 Ge (200 g/L) 103 Rh and 185 Re (50 g/L)

**Table 2:** The values of the q and RfD of model parameters.

Carcinogenic Chronic Chemical Pollutants	Q	Non-Carcinogenic Chronic Chemical Pollutants	RfD
As	15	Cu	5.0 × 10^−3^
Cd	6.1	Zn	3.0 × 10^−4^
Cr	41	Hg	1.0 × 10^−4^
		Pb	1.4 × 10^−3^
		Ni	2 × 10^−2^
		Se	5 × 10^−3^

Unit of the Q: [mg·(kg·d)^−1^]; Unit of the Q RfD: [mg·(kg·d)^−1^].

**Table 3:** The maximum risk acceptance risk level and negligible level of some organizations.

Organization	Maximum Acceptable Risk Level/a^−1^	Risk Level that Can be Ignored/a^−1^	Remarks
U.S. Environmental Protection Agency	1 × 10^−4^	-	Radical
International Commission on Radiological Protection	5 × 10^−5^	-	-
Royal Society	1 × 10^−6^	1 × 10^−7^	-
Holland Construction and Environment Department	1 × 10^−6^	1 × 10^−8^	Chemical pollutants
Swedish Environmental Protection Agency	1 × 10^−6^	-	Chemical pollutants

**Table 4:** Descriptive statistics analysis of the trace elements in the drinking water of the Bay Xinjiang China.

Study Area	Water Source	Element	Min	Max	Mean	SD	CV
DW	The well water	Cd	0.01	0.138	0.031	0.034	1.067
		As	0.716	8.008	2.283	2.023	0.886
		Cr	1.796	8.28	3.79	1.868	0.493
		Zn	0.517	17.143	4.46	5.42	1.21
		Ni	0.537	1.824	0.862	0.432	0.50
		Pb	0.004	0.13	0.04	0.04	1.07
		Se	0.66	3.787	1.466	1.066	0.727
KYR	Fountain water	Cd	0.005	0.042	0.01617	0.0089	0.212
		As	0.302	8.964	1.4959	2.1919	0.244
		Cr	1.592	4.722	2.5243	0.7267	0.154
		Zn	0.467	5.183	1.7802	1.240	0.239
		Ni	0.624	1.901	1.2216	0.3810	0.2004
		Pb	0.006	0.121	0.0454	0.0392	0.324
		Se	0.305	1.333	0.7855	0.365	0.274
YTR	Fountain water	Cd	0.003	0.016	0.0078	0.00434	0.556
		As	0.326	0.472	0.3915	0.0405	0.1035
		Cr	1.809	4.862	2.53340	1.01244	0.3999
		Zn	0.955	24.97	6.9017	7.266	1.0527
		Ni	1.475	1.923	1.7457	0.1274	0.072
		Pb	0.002	0.023	0.0103	0.00737	0.716
		Se	5.68	9.053	7.2455	1.179	0.162
KRW	Mountain melting glacier water	Cd	0.013	0.127	0.03369	0.02314	0.686
		As	0.522	5.728	1.5786	1.333	0.8445
		Cr	0.784	2.133	1.4382	0.2806	0.1951
		Zn	0.395	449.048	56.233	115.60	2.055
		Ni	0.721	1.73	0.9497	0.2066	0.217
		Pb	0.007	0.466	0.0719	0.0879	1.221
		Se	0.188	0.796	0.5553	0.1319	0.2375
Y	Mountain melting glacier water	Cd	0.011	0.025	0.0175	0.005167	0.295
		As	0.466	0.5501	0.5030	0.03417	0.0679
		Cr	0.876	1.158	0.9725	0.10908	0.112
		Zn	6.865	349.639	96.189	132.75	1.380
		Ni	0.674	0.806	0.7063	0.04999	0.070
		Pb	0.001	0.009	0.00516	0.0026	0.5108
		Se	0.616	1.104	0.7885	0.1668	0.2116
B	Fountain water	Cd	0.008	0.022	0.0153125	0.00457	0.298
		As	0.246	0.451	0.355	0.0606	0.1705
		Cr	1.158	3.056	1.737	0.6017	0.346
		Zn	0.49	6.835	1.9063	1.946	1.021
		Ni	1.012	1.459	1.311	0.1043	0.079
		Pb	0.002	0.032	0.007375	0.00683	0.926
		Se	2.577	4.565	3.635	0.6233	0.171

DW: Dawanqi; KYR: Keyiri; KRW: Kriwahi; Y: Yangchang; YT: Yaturi; B: Bulong. CV: Coefficient of Variation; SD: Standard deviation.
