# Correction: TURDI, M. et al. Trace Elements Contamination and Human Health Risk Assessment in Drinking Water from the Agricultural and Pastoral Areas of Bay County, Xinjiang, China; *Int. J. Environ. Res. Public Health* 2016, *13*, 938

**DOI:** 10.3390/ijerph15040705

**Published:** 2018-04-10

**Authors:** Muyessar Turdi, Linsheng Yang

**Affiliations:** 1 Key Laboratory of Land Surface Pattern and Simulation, Institute of Geographical Sciences and Natural Resources Research, Chinese Academy of Sciences, 11 A Datun Road, Beijing 100101, China; muys.14b@igsnrr.ac.cn; 2 University of Chinese Academy of Sciences, Beijing 100049, China

In the original version of our article [1], the Formula (3) was written $R_{ig}^{c}=\frac{1\times \exp(ADD_{ig}\times q_{ig})}{Y}$.

However, the formula should be $R_{ig}^{c}=\frac{1-exp\left(-ADD_{ig}\times q_{ig}\right)}{Y}$. During the data analysis process we used the right formula so the data analysis result is right, so we only need make revision to the third formula.
